# Transcriptomics and Proteomics Analyses of the Responses of *Propionibacterium acidipropionici* to Metabolic and Evolutionary Manipulation

**DOI:** 10.3389/fmicb.2020.01564

**Published:** 2020-08-13

**Authors:** Tingting Liu, Qianru Zhao, Yang Li, Liying Zhu, Ling Jiang, He Huang

**Affiliations:** ^1^College of Biotechnology and Pharmaceutical Engineering, Nanjing Tech University, Nanjing, China; ^2^College of Food Science and Light Industry, Nanjing Tech University, Nanjing, China; ^3^College of Chemical and Molecular Engineering, Nanjing Tech University, Nanjing, China; ^4^College of Pharmaceutical Science, Nanjing Tech University, Nanjing, China

**Keywords:** *Propionibacterium acidipropionici*, propionic acid, metabolic engineering, evolutionary engineering, multiple stress, omics analyses

## Abstract

We first performed a combination of metabolic engineering (deletion of *ldh* and *poxB* and overexpression of *mmc*) with evolutionary engineering (selection under oxygen stress, acid stress and osmotic stress) in *Propionibacterium acidipropionici*. The results indicated that the mutants had superior physiological activity, especially the mutant III obtained from *P. acidipropionici-*Δ*ldh*-Δ*poxB*+*mmc* by evolutionary engineering, with 1.5–3.5 times higher growth rates, as well as a 37.1% increase of propionic acid (PA) titer and a 37.8% increase PA productivity compared to the wild type. Moreover, the integrative transcriptomics and proteomics analyses revealed that the differentially expressed genes (DEGs) and proteins (DEPs) in the mutant III were involved in energy metabolism, including the glycolysis pathway and tricarboxylic acid cycle (TCA cycle). These genes were up-regulated to supply increased amounts of energy and precursors for PA synthesis compared to the wild type. In addition, the down-regulation of fatty acid biosynthesis and fatty acid metabolism may indicate that the repressed metabolic flux was related to the production of PA. Quantitative reverse-transcription polymerase chain reaction (qRT-PCR) was performed to verify the differential expression levels of 16 selected key genes. The results offer deep insights into the mechanism of high PA production, which provides the theoretical foundation for the construction of advanced microbial cell factories.

## Introduction

Propionic acid (PA), a potential building block for C3-based bulk chemicals, is used as a food preservative and antifungal agent because of the antimicrobial properties of its calcium-, potassium-, and sodium salts, as well as in the manufacture of pharmaceuticals, perfumes, pesticides and fungicides (Liu et al., 2012). Currently, the traditional fossil-fuel-based PA synthesis is becoming increasingly less desirable due to energy shortages, environmental pollution and the desire for sustainable development, while the fermentation of PA from renewable resources using *Propionibacterium* has unique advantages (Guan et al., 2015). As the major production strains, the members of the genus *Propionibacterium* are generally recognized as safe (Guan et al., 2015). *P. acidipropionici*, *P. shermanii* and *P. freudenreichii* are capable of utilizing a wide range of carbon sources to produce PA, including corn stover hydrolysate (Wang et al., 2017), sorbitol (Duarte et al., 2015), glycerol and glucose (Zhang et al., 2015) and whey lactose (Jiang et al., 2015). To date, many strategies have been developed to improve the yield of PA, including high density fermentation (Wang et al., 2015a), immobilization of *Propionibacterium* cells (Wallenius et al., 2015), introduction of biocompatible small molecule (Jiang et al., 2015), reduction of by-product (Zhang and Yang, 2009), controlled pH-shift in fed-batch culture (Xin et al., 2014) and engineering of metabolic pathways (Liu et al., 2016). However, in addition to the common problems of industrial fermentation such as end-product inhibition and by-product accumulation, the anaerobic growth of *Propionibacterium* spp. means that the culture conditions for PA production must be strictly controlled (Aburjaile et al., 2016). Although the creation of an anaerobic environment is no longer as challenging due to the development of production technology, the cost of maintaining anaerobic conditions is high and incompatible with the concept of developing a green economy. PA is synthesized by *Propionibacterium* spp. via the dicarboxylic acid pathway, which also generates byproducts such as lactic acid (LA) and acetic acid (AA) (Zhang and Yang, 2009). Thus, the accumulation of acid products during fermentation easily results in an acidic environment, affecting cell activity and inhibiting metabolism, which precludes industrial-scale PA production. Moreover, hypertonic solutions caused by higher substrate concentration tend to dehydrate cells and inhibit their growth and reproduction (Purvis et al., 2005; Fernandes et al., 2008). Consequently, enhancing the tolerance of *Propionibacterium* spp. to oxygen, acid and hypertonic solutions is considered as an effective strategy to alleviate the inhibition and has triggered a research hotspot.

Metabolic engineering has been used as an effective strategy for increasing PA production. Liu et al. (2016) improved the PA titer of *P. jensenii* by 34.7% by overexpressing phosphoenolpyruvate carboxylase (*ppc*) and deleting lactate dehydrogenase (*ldh*). Guan et al. (2018) obtained a 12.2% increase of PA by constructing a *P. acidipropionici* strain with simultaneous deletions of *ldh*1, *ldh*2 and *pox*B. Evolutionary engineering, also defined as adaptive laboratory evolution, is a popular approach to develop high-performing strains for industrial application (Mans et al., 2018). Kato et al. (2017) developed evolutionary engineering of *Chlamydomonas* sp. by adding a certain amount of sea salt to enhance significantly biomass production. Furthermore, Jantama et al. (2008) performed combining metabolic and evolution engineering of *Escherichia coli* C to obtain the high yield of succinate and malate. However, combining metabolic engineering with laboratory evolution to improve the synthesis of PA has not been published. Omics technologies are able to identify crucial genes and metabolites, providing valuable information that reflects stress-induced changes and the intricate interplay between organisms and the environment (Huang et al., 2018; Meng et al., 2019). For example, comparative genomic and transcriptomic analyses of *P. acidipropionici* revealed the molecular mechanisms of acid tolerance, while proteomics analyses revealed how *P. acidipropionici* respond to PA stress (Guan et al., 2014).

Therefore, in this study, *P. acidipropionici* CGMCC 1.2232 was first engineered by combining metabolic engineering with evolutionary engineering to obtain a superior strain defined by strong robustness and high PA production capacity. Fermentation analysis showed that a 37.1% increase of PA titer and a 37.8% increase of PA productivity was obtained in the mutant III. The integrated transcriptomic and proteomic analyses suggested that several differentially expressed genes (DEGs) and proteins (DEPs) in the mutant III involved in central carbon metabolism were up-regulated, while those related to fatty acid biosynthesis were down-regulated. Moreover, the expression changes of 16 genes involved in central carbon metabolism were verified using qRT-PCR. These results allow a more comprehensive view of the mechanism of high PA production.

## Materials and Methods

### Microorganisms and Culture Media

All strains, plasmids and primers used in this study are listed in Supplementary Table S1. *P. acidipropionici* CGMCC1.2232 was propagated anaerobically at 30°C in culture medium containing the following: yeast extract 5 g⋅L^–1^; peptone 5 g⋅L^–1^; dipotassium hydrogen phosphate 0.25 g⋅L^–1^; manganese sulfate 0.05 g⋅L^–1^; glucose 5 g⋅L^–1^. Resazurin was added to a final concentration of 0.05 % as an oxygen indicator. *Escherichia coli* DH5α was used for cloning and plasmid propagation, and was grown aerobically in Luria-Bertani (LB) medium at 37°C. *E. coli* S17–1 (λ pir) was used as the donor strain to transform *P. acidipropionici* via conjugation. All media were sterilized through autoclaving (121°C for 20 min), after which 50 μg⋅mL^–1^ ampicillin, kanamycin, and erythromycin or 25 μg⋅mL^–1^ spectinomycin was added when necessary.

### Genetic Manipulation and Evolutionary Engineering

The chromosomal target genes were deleted seamlessly according to the method we reported previously (Jiang et al., 2015). The upstream and downstream regions flanking the lactate dehydrogenase (*ldh*) and pyruvate oxidase (*pox*B) genes were amplified using the genome of *P. acidipropionici* as the template. The resulting products with the 2.0 kb Smr/Spcr Ω cassette were amplified using the high copy vector pBluescriptII SK+ (Stratagene). The Kan^R^-suicide vector pJP5603 was selected to construct the recombinant plasmids pJLDH and pJPOXB that were transferred into *P. acidipropionici* cells by conjugation using the donor strain *E. coli* S17–1 (λ pir). The overexpression of genes was conducted as described before (Jiang et al., 2015). The methylmalonyl-CoA carboxyltransferase (*mmc*) gene was amplified from the genome of *P. acidipropionici* using the primers *mmc*-for1 and *mmc*-rev1. The shuttle vector pBRESP36A was selected to construct the *mmc* overexpression plasmid pBMMC that was transferred into *P. acidipropionici* via conjugation.

Evolutionary engineering of *P. acidipropionici* mutants was carried out using multiple stress in the form of different pH values (7, 6, 5, 4), glucose concentrations (60, 90, 120 g⋅L^–1^) and oxygen flux values (99.999% N_2_, 79% N_2_ + 11% CO_2_ + 10% O_2_, 79% N_2_ + 21% O_2_) during anaerobic cultivation in a 2 L NBS fermenter at 30 °C and 120 rpm (Siero et al., 2015; Wu et al., 2017). Samples were taken every 24 h to assess cell growth and stress tolerance. Fresh medium was added to maintain a normal 2 L continuous culture system. multiple stress was applied stepwise to maintain the cells at an OD_600_ above 1.0. The final mutant was then used as the experimental group, and the wild-type was considered as the control group. The two groups were cultured under single stress (pH, glucose concentration, oxygen flux) to evaluate cell growth and compare their stress tolerance.

### Batch Fermentation of PA Using the Engineered *P. acidipropionici*

For PA fermentation, strains were pre-cultured in 100 mL anaerobic bottles with 50 mL medium with different pH, glucose concentrations and oxygen flux until the OD_600_ reached 2.0. Then, 5% of seed liquid was used to inoculate a 2-L NBS fermenter with corresponding medium, followed by anaerobic culture at 30°C and 120 rpm for batch fermentations. Samples were taken regularly to measure the concentrations of LA, AA and PA. Additionally, the wild type and the evolved mutants were subjected to transcriptomic and proteomic analyses to further investigate the tolerance mechanism of *P. acidipropionici*.

### RNA Sequencing and Statistical Analyses

Total RNA of the wild type and the mutant III obtained via evolutionary engineering from *P. acidipropionici-*Δ*ldh*-Δ*poxB*+*mmc* were extracted using an RNeasy Mini Kit (Qiagen, Hilden, Germany). The concentration and purity of the RNA was measured using a NanoDrop instrument. Then, mRNA was isolated and purified using the Poly (A) Purist^TM^ MAG Kit (Ambion, United States). The cDNA of the transcriptomes was synthesized using the SMARTer^TM^ PCR cDNA Synthesis Kit (Clontech, United States). The NEBNext^®^ mRNA Library Prep Reagent Set for Illumina^®^ (NEB, UAS) was performed to construct a cDNA library (∼300 bp), which was subsequently sequenced on the Illumina Xten platform (PE150 mode).

The clean data collected after filtering was aligned to the reference transcript sequences using SOAP2. The alignment was conducted via RPKM conversion to obtain the expression level of the transcript.

(1) $$\mathrm{RPKM}=\frac{\mathrm{total}\,\mathrm{exon}\,\mathrm{reads}}{\mathrm{mapped}\,\mathrm{reads}\,\left(\mathrm{millions}\right)*\mathrm{exon}\,\mathrm{length}\,\left(\mathrm{KB}\right)}$$

Differential expression genes were analyzed using the R package edgeR, and the screening threshold was *p*-value < 0.05, log FC (fold change (condition 2/condition 1) for a gene) ≥ 1 or log FC ≤ −1.

### Functional Enrichment Analyses of the Differentially Expressed Genes

Gene Ontology analysis of the DEGs was performed using a hyper-geometric distribution. Each GO term with false discovery rate (FDR) ≤ 0.05 was chosen as a significant enriched GO entry. Significant enrichment by pathway can determine the most important biochemical metabolic pathways and signal transduction pathways related to the DEGs. KEGG (Kyoto Encyclopedia of Genes and Genomes) classification was analyzed using R software by setting the parameter-fdr to BH to find pathways (FDR ≤ 0.05) where differential genes are significantly enriched relative to all annotated genes.

(2) $$p=1-\sum_{i-0}^{m-1}\frac{\left(\begin{array}{c} M\\ i \end{array}\right)\,\left(\begin{array}{c} N-M\\ n-i \end{array}\right)}{\left(\begin{array}{c} N\\ n \end{array}\right)}$$

where N is the number of genes with pathway annotation; n is the number of differentially expressed genes in N; M is the number of genes annotated as belonging to a particular pathway; m is the number of differentially expressed genes in M.

### Protein Extraction and LC-MS/MS Analysis

A high strength ultrasonic processor (Scientz, China) was used to treat the samples on ice three times in the lysis buffer (8 M urea, 1% protease inhibitor mixture). The cell debris was removed by centrifugation at 12,000 *g* and 4 °C for 10 min, and the supernatant was collected. The protein concentration was determined using a BCA kit according to the manufacturer’s instructions. The peptide obtained by trypsin digestion was reconstituted in 0.5 M Triethylammonium Bicarbonate (TEAB) and processed by a TMT kit/iTRAQ kit (AB Sciex, Foster City, CA, United States). The tryptic peptides resuspended in 0.1% formic acid (buffer A) were directly loaded onto a self-made reversed-phase analytical column (15 cm length, 75 μm inner diameter). The gradient consisted of 6-23% buffer B (0.1% formic acid in 98% acetonitrile) over 26 min, 23–35% in 8 min, 35–80% in 3 min, then hold at 80% for the last 3 min before reversing to buffer A for re-equilibration. The flow rate was set at constant 400 nL⋅min^–1^ on an EASY-nLC 1000 UPLC system.

The peptides were exposed to a nano-spray ionization (NSI) source followed by tandem mass spectrometry (MS/MS) in a Q Exactive^TM^ Plus instrument (Thermo Fisher Scientific, United States) coupled online to the UPLC. The applied electrospray voltage was 2 kV. The m/z scan range was from 350 to 1,800 for full scan, and whole peptides were detected at 70,000 resolution in the OrbiTrap. Peptides were then selected for MS/MS using an NCE setting of 28, and the fragments were detected at 70,000 resolution in the OrbiTrap. A data-dependent program was performed alternately after an MS scan, followed by 20 MS/MS scans, and eliminated dynamically after 15 s. Automatic gain control (AGC) was set at 5E4. The first fixed mass was set as 100 m/z.

### Data Processing and Functional Enrichment Analyses of Differentially Expressed Proteins

The MaxQuant search engine (v.1.5.2.8) was used to analyze the obtained MS/MS data. Tandem mass spectra were searched against the UniProt Proteome database of *P. acidipropionici* concatenated with a reverse decoy database. Trypsin/P was designated as the enzyme, allowing up to two missed cleavage sites. The mass tolerance of precursor ions was set to 20 ppm in the first retrieval, 5 ppm in the main retrieval and 0.02 Da for fragment ions. Oxidation of methionines was designated as variable modification, while carbamidomethylation of cysteines was designated as a fixed modification. The FDR for modification sites, peptides and proteins was adjusted to < 1% and the lowest score for peptides was set at > 40. The DEPs were selected when the difference ratio of mutant III/wild type was greater than 1.5 (significantly up-regulated proteins) or less than 1.15 (significantly down-regulated proteins) with a *t*-test *p*-value < 0.05.

The proteins were classified into three categories: biological process, cellular component and molecular function by GO annotation^1^. Two-tailed Fisher’s exact test was used to test the enrichment of the DEPs based on all identified proteins from the transcriptome of *P. acidipropionici*. The *p*-value from the test was subjected to negative logarithmic (−log10) conversion. Moreover, KEGG^2^ analysis was performed to predict enriched pathways in which the DEPs were involved using the uniform two-tailed Fisher’s exact test. KEGG mapper was used to map the annotation results onto the KEGG pathway database. These pathways were classified into hierarchical categories according to the KEGG website. Items with a corrected *p*-value < 0.05 were considered significant.

### qRT-PCR Determination

The qRT-PCR experiment was carried out in QuantStudio 3 and 5 Real-Time PCR Systems and Applied Biosystems^TM^ TaqMan^TM^* (Thermo Scientific, Wilmington, DE, United States) as previously reported (Guan et al., 2014). Cells of wild type and the mutant III were cultured overnight and then transferred into fresh liquid medium to control OD_600_ at ∼0.6. Total cellular RNA was extracted by using the RNeasy Mini Kit (Qiagen, Germany). cDNA was synthesized using a PrimeScript RT reagent Kit With gDNA Eraser (Takara, Dalian, China). The primers of verification genes were designed by using Primer Express 3.0 (Supplementary Table S2) and followed with PCR amplification (pre-incubation at 95°C for 30 s; 40 cycles at 95°C for 5 s, 60°C for 20 s, and cooling at 50°C for 30 s). The 20.0 μL reaction system was composed of 10.0 μL SYBR Premix Ex Taq (2×), 1.2 μL DNA template, 0.4 μL forward and reverse primers (10 μM), and 8 μL dH_2_O. The rimM, encoding 16S rRNA processing protein, was selected as a reference gene for normalization.

### Analytical Methods

The optical density at 600 nm (OD_600_) was measured using a standard spectrophotometer (Ultrospec 3300 pro, Amersham Bioscience) to analyze cell growth. The concentrations of organic acids were determined by high-performance liquid chromatography (HPLC) on an Aminex HPX-87H ion-exclusion column (Bio-Rad, United States, 9 μm × 300 mm × 7.8 mm) and ERC 7515A refractive index detector (ERC, Saitama, Japan). 10 mM H_2_SO_4_ solution at a flow rate of 0.4 mL/min was used as a mobile phase. The column temperature was set at 55°C and refractometer temperature was set at 30°C. Samples were centrifuged at 12,000 *g* for 5 min followed by dilution in ultrapure water and then boiled for 15 min. The supernatant obtained by centrifugation at 13,000 *g* for 15 min was filtered through 0.22 μm membrane pore size (diameter, 25 mm) for analysis.

## Results and Discussion

### The Growth Profiles of Mutant Strains Through Metabolic and Evolutionary Engineering

**FIGURE 1:**
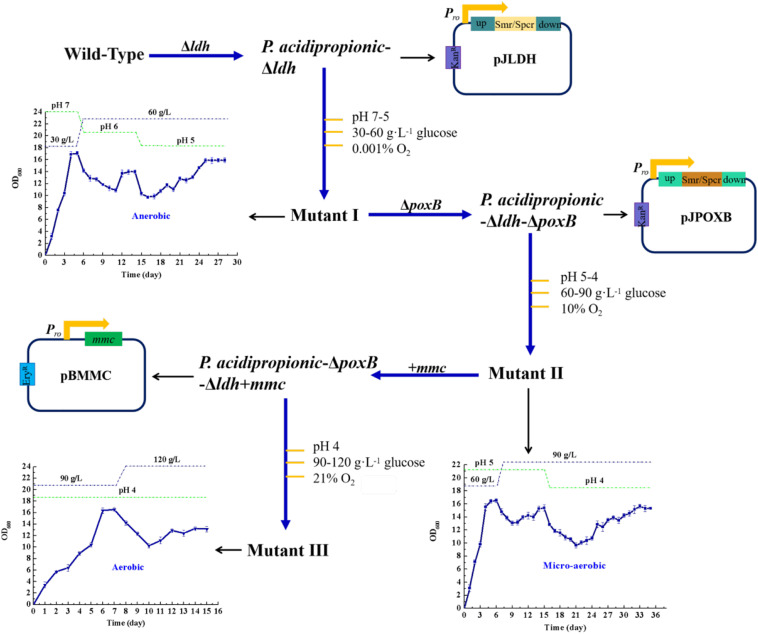
A schematic overview of the experimental setting in regard to metabolic and evolutionary engineering of *P. acidipropionici*.

The *ldh* and *poxB* genes, which are main contributors of the generation of the by-products acetate and lactate (Liu et al., 2016), were deleted. Additionally, the *mmc* gene from *P. acidipropionici*, which controls the carbon flow into the propionate-producing Wood-Werkman cycle (Wang et al., 2015b), was overexpressed. These manipulations yielded the strains *P. acidipropionici-*Δ*ldh, P. acidipropionici-*Δ*ldh*-Δ*poxB*, and *P. acidipropionici-*Δ*ldh*-Δ*poxB*+*mmc*, respectively. In evolutionary engineering, specific stress is applied gradually on the basis of the growth rate of the adapted strain, which gives a buffer period so that the strain can adequately mobilize the *in vivo* response mechanisms to resist stress (Figure 1). During the evolution of *P. acidipropionici-*Δ*ldh* into the mutant I, the growth of the experimental strain displayed a peak at OD_600_ 17.1 on the fourth day of culture, followed by a downward trend because of the decline of pH and the increase of glucose concentration. Subsequently, the growth rate of *P. acidipropionici-*Δ*ldh* tended to be stable at OD_600_ 13.9 and began to descend when the pH was decreasing again (6 to 5), and was ultimately stable at OD_600_ 15.9. From *P. acidipropionici-*Δ*ldh*-Δ*poxB* to the mutant II (deletion of *poxB* followed by evolutionary engineering), the formation of the subsequent two peaks at OD_600_ 16.5 and 15.4 on the 6th and 15th day corresponded to the progressive deepening of the levels of single stress factors. From *P. acidipropionici-*Δ*ldh*-Δ*poxB*+*mmc* to the mutant III (overexpression of *mmc* followed by evolutionary engineering), the growth of the strains started to pick up a steady upward trend, and the strain showed a normal growth curve after supplementation with fresh medium. The highest OD_600_ was 14.6, while the lowest OD_600_ of 9.8 was observed with pH 4, 120 g⋅L^–1^ glucose and 21% oxygen sparging. These results indicate that the experimental strain could still maintain a considerable growth rate under the superimposed multiple stress, even when exposed to simultaneously increasing stress levels. This indicated that the strain had developed a certain level of ‘multiple-stress’ protection and acquired a fitness advantage based on the gradual application of stress by artificial control so that it could deal with multiple stress.

To further investigate the growth rate of the strain obtained by metabolic and evolutionary engineering under multiple stress, the wild type and mutant III were compared. Compared with the 0.09 h^–1^ specific growth rate of the wild type under normal fermentation conditions (pH 7), mutant III had a significant improvement at 0.14 h^–1^, which was apparently higher than the 0.131 ± 0.007 h^–1^ previously reported for growth on soy molasses and soy molasses hydrolysate (Yang et al., 2018). However, when the strains were exposed to low pH, the growth of both the wild type and the mutant III was suppressed accordingly. Nevertheless, the growth of the mutant III was significantly superior to that of the wild type. With the decrease of pH, the growth rate difference between the wild type and mutant III gradually increased, and mutant III could maintain normal growth, while the growth of the wild type was clearly suppressed (Supplementary Figure S1A). As the concentration of glucose in the medium increased, the difference of the growth rate between the wild type and mutant III gradually increased. At normal glucose concentrations (30 g⋅L^–1^ glucose), the specific growth rate of the wild type was about 0.09 h^–1^, while that of mutant III reached 0.2 h^–1^, which was 1.4-fold higher than the previously reported value for the same glucose concentrations (Wang et al., 2015a). While the wild type almost stopped growing at a high glucose concentration of 120 g⋅L^–1^, mutant III could maintain growth at OD_600_ 0.4 (Supplementary Figure S1B). Furthermore, the strain exhibited similar growth characteristics when exposed to oxygen stress compared with acid stress and osmotic stress. A 2-fold growth rate difference has been observed under anaerobic fermentation conditions (oxygen content 0.001%). Under these conditions, the specific growth rate of mutant III reached 0.18 h^–1^, while that of the wild type was 0.09 h^–1^. By contrast, with 21% oxygen content in the sparged gas mixture, the specific growth rate of the wild type was only 0.03 h^–1^, while that of mutant III was 0.05 h^–1^ (Supplementary Figure S1C). As a result, the growth performance of mutant III under multiple stress was clearly superior to that of the wild type. Importantly, the generation of the mutant III has proved that evolutionary engineering is an effective method for improving the resistance of *P. acidipropionici* to multiple stress.

### PA Fermentation Using the Engineered *P. acidipropionici*

Batch fermentations were carried out using wild-type *P. acidipropionici* as well as the mutants I, II and III to investigate their PA production capacity. As shown in Table 1, under the optimal culture conditions (pH 7, 99% N_2_, 5 g⋅L^–1^ glucose), the PA titer and productivity of mutant I were increased by 12.4 and 13.5% compared to the wild type. Its performance was therefore also significantly better than that of *P. jensenii*-Δ*ldh* with 1.6 and 8.1% enhancement (Liu et al., 2016), respectively. At the same time, a 68.8% of decrease of LA yield (3.06 ± 0.82 g⋅L^–1^ vs. 1.04 ± 0.17 g⋅L^–1^) was obtained. However, there was a 3% increase of AA titer (5.24 ± 0.83 g⋅L^–1^ vs. 5.63 ± 0.62 g⋅L^–1^), which may be related to the deletion of *ldh*, which increased carbon flow from pyruvate into the synthesis of LA (Luna-Flores et al., 2018). The PA production of the mutant II only increased by 3.2%, while the titers of AA and LA were both decreased by more than 70%, which was consistent with the variation tendency of the PA, LA and AA titers of *P. acidipropionici*-Δ*poxB*-Δ*ldh* (Guan et al., 2018). A further 37.1% increase of PA titer (28.1 ± 0.96 g⋅L^–1^ vs. 38.7 ± 1.14 g⋅L^–1^) and 37.8% increase of PA productivity (0.216 ± 0.006 g⋅L^–1^ vs. 0.298 ± 0.008 g⋅L^–1^⋅h^–1^) was achieved in the mutant III. This increase was much more significant than the reported value achieved via the overexpression of *mmc* in *P. freudenreichii* in batch fermentation (Wang et al., 2015b). At the same time, there was a 78.6 and 87.8% decrease of the byproduct yields because of the deletion of *ldh* and *poxB*, and the overexpression of *mmc* drove the carbon flow into the synthesis of PA to the greatest extent.

**TABLE 1:** Analysis of microbial production using engineered *P. acidipropionici* in batch fermentation.

	PA titer	LA titer	AA titer	PA productivity
Strain	(g⋅L^–1^)	(g⋅L^–1^)	(g⋅L^–1^)	(g⋅L^–1^⋅h^–1^)
WT	28.1 ± 0.96	3.06 ± 0.82	5.24 ± 0.83	0.216 ± 0.006
Mutant I	31.8 ± 0.85	1.04 ± 0.17	5.63 ± 0.62	0.245 ± 0.007
Mutant II	29.4 ± 0.59	0.89 ± 0.06	0.78 ± 0.04	0.226 ± 0.006
Mutant III	38.7 ± 1.14	0.78 ± 0.05	0.69 ± 0.05	0.298 ± 0.008

### Transcriptomics Analyses Between the Wild Type and the Mutant III

#### Illumina HiSeq mRNA Sequencing

The adaptation of microbes to environmental stress and genetic manipulation can produce high-performance phenotypes through subsequent positive selection (Almario et al., 2013). However, in this study, the molecular basis has not been clarified. Based on this, to discern the overview of gene expression profiles of multiple stress and genetic manipulation on *P. acidipropionici*, the transcriptome analysis of the wild type and the mutant III was carried out. Overall, a total of 13.43 and 11.68 million reads of the wild type and the mutant III were, respectively, detected by SOAP2 based on the NCBI library. The differences in gene expression between the wild type and the mutant III were investigated. A total of 166 genes with different expression levels (*p* < 0.05) were identified in the mutant III from uniquely matched reads data, with 72 up- and 94 down-regulated genes compared to the wild type. The identified 166 DEGs were annotated using the GO database to explore the biological functions. As can be shown in Supplementary Figure S2, DEGs involved in the categories metabolic process, single-organism process in biological process, membrane and membrane part in cellular component, catalytic activity, binding and transporter activity in molecular function were all enriched by choosing FDR value less than 0.05 as a significant threshold in the mutant III compared to the wild type. This result of GO analysis suggested that the DEGs involved in these items were positively responsive, especially the major annotated membrane part and catalytic activity, which were most significantly affected by the multiple stress and genetic manipulation. All predicted DEGs were mapped to the related pathways in the KEGG database. The top 20 enriched KEGG pathways were displayed in the scatter plot (Supplementary Figure S3). Among them, the three most significantly enriched pathways were also mapped, including fatty acid biosynthesis, fatty acid metabolism and biotin metabolism (corrected *p*-value < 0.01).

#### Differentially Expressed Genes (DEGs) Involved in ABC Transporters and Quorum Sensing

To maintain the equilibrium conditions necessary for cell survival under stress, the transport of various substrates such as sugars, amino acids, peptides and ions is required, which is accomplished by ABC transporters present in the cell membrane (Zhu et al., 2019). In this study, we paid particular attention to the differentially expressed genes related to the most enriched ABC transporters under multiple stress and genetic manipulation. We found that the genes *msmF* and *msmG* responsible for the utilization of various carbohydrates in the mutant III, which encode melibiose transport system permease, were, respectively, significantly up-regulated 8.03- and 8.89-fold compared to the wild type (Table 2). The genes *cebF* (cellobiose transport system permease protein) and *cycB* (maltooligosaccharide transport system substrate-binding protein) show 2.98- and 2.36-fold up-regulation in the mutant III. For microorganisms under abiotic stress, the acquisition and metabolism of carbohydrates is essential for survival. To achieve optimal utilization of carbohydrates, microbial cells carried out appropriate adjustment of the metabolism and gene expression patterns (Lemos et al., 2008). The above result indicated that the engineered strain may have developed a self-regulatory mechanism to achieve optimal flow of carbohydrates and their metabolism. Moreover, three gene related to ribose transport (*rbsA*, *rbsB* and *rbsC*) were 3. 21-, 4.12- and 3.55-fold up-regulated in the mutant III, respectively. The ribose transporter is mainly committed to the uptake of ribose. The high up-regulations of the *rbsA* and *rbsB* genes in the *rbs* operon implied that the transmitting of molecular precursors for nucleic acid synthesis was more active compared to the wild type. Interestingly, the gene *ABC.FEV.P* encoding iron complex ABC transporter permeases showed dramatic 6.96-fold down-regulation, which may be related to the homeostatic regulation of substrates in *P. acidipropionici.*

**TABLE 2:** Differential expression genes related to ABC transporters and quorum sensing.

Metabolic pathways	Entry	Gene name	Definition	MutantIII/Wildtype fold change
ABC transporters	K10118	*msmF*	melibiose transport system permease protein	8.03↑^∗^
	K10119	*msmG*	melibiose transport system permease protein	8.89↑^∗^
	K10241	*cebF*	cellobiose transport system permease protein	2.98↑
	K15770	*cycB*	maltooligosaccharide transport system substrate-binding protein	2.36↑
	K02013	*ABC.FEV.A*	iron complex transport system ATP-binding protein	6.96↓^∗^
	K02015	*ABC.FEV.P*	iron complex transport system permease protein	3.96↓
	K16787	*ecfA2*	energy-coupling factor transport system ATP-binding protein	4.28↓
	*K16785*	*ecfT*	energy-coupling factor transport system permease protein	5.86↓^∗^
	K10441	*rbsA*	ribose transport system ATP-binding protein	3.21↑
	K10439	*rbsB*	ribose transport system substrate-binding protein	4.12↑
	K10440	*rbsC*	ribose transport system permease protein	3.55↑
Quorum sensing	K02035	*ABC.PE.S*	nickel transport system substrate-binding protein	3.81↓
	K02033	*ABC.PE.P*	nickel transport system permease protein	3.17↓
	K02034	*ABC.PE.P1*	nickel transport system permease protein	3.08↓
	K01897	*ACSL*	long-chain acyl-CoA synthetase	2.85↓

*↑ upregulation, ↓ downregulation. ^∗^Fold change was significantly higher than 5.*

In addition to the common stress-response mechanisms mediated by ABC transporters, we also observed transcriptional differences of the genes related to quorum sensing, which was identified as one of the important contributors to the coordination of behavior among cells, in the two strains (Asfahl and Schuster, 2018). Three genes involved in nickel transport (*ABC.PE.S*, *ABC.PE.P* and *ABC.PE.P1*) showed identical expression patterns, with about 3-fold down-regulation in the mutant III. Meanwhile, the gene *ACSL* encoding long-chain acyl-CoA synthetase, which transports fatty acids from the periplasm to the cytosol at the expense of ATP, exhibited 2.85-fold down-regulation compared to the wild type (Weimar et al., 2002).

### Proteomics Analysis Between the Wild Type and the Mutants III

#### Protein Identification and Annotation

To identify differences protein abundance, the proteomics research between the wild type and the mutant III was carried out (Guan et al., 2014). A total of 2,158 proteins were identified in the proteomic analysis. There were 343 differentially expressed proteins (DEPs) in the mutant III, with 217 up- and 126 down-regulated proteins. To further determine the DEPs more likely to be mobilized to resist stress, we enriched the DEPs. The GO enrichment annotation shown in Supplementary Figure S4 classified the majority of DEPs into oxidation-reduction process, carbohydrate metabolic process in biological process, oxidoreductase activity, glutathione transferase activity in molecular function in the mutant III, which was hardly consistent with the GO enrichment analysis of the DEGs. This result suggested the regulation of functional genes in response to adverse environmental conditions and genetic manipulation in the mutant III which led to changes in the mRNA and protein expression levels. The KEGG pathway enrichment (Supplementary Figure S5) indicated that the top KEGG pathways for the proteins that were up-regulated were TCA cycle and carbon metabolism, while the significant pathways detected for the proteins that were downregulated were ribosome, nitrogen metabolism and fatty acid biosynthesis.

#### Differentially Expressed Proteins Involved in Central Carbon Metabolism

Central carbon metabolism converts carbohydrates into metabolic precursors by utilizing a complicated range of enzyme reactions, which in turn promote the biomass accumulation (Minic, 2015). Thus, central carbon metabolism is indispensable for cell growth and metabolism. In this study, proteomic analysis revealed that the expression of 12 proteins in the mutant III related to central carbon metabolism composed of the glycolytic pathway, pentose phosphate (PP) pathway and TCA cycle were up-regulated compared to the wild type. As shown in Table 3, the majority of proteins that were observed are part of the TCA cycle. Compared to the wild type, various proteins in the mutant III participating in the TCA cycle including 2-oxoglutarate dehydrogenase E1 component and E2 component (*sucA*, *sucB*), pyruvate:ferredoxin (flavodoxin) oxidoreductase (*porA*), and citrate synthase (*gltA*), succinate dehydrogenase flavoprotein subunit (*sdhA*) were obviously up-regulated, especially *porA* and *sdhA* with 3.32- and 2.93-fold up-regulation. In addition, pyruvate phosphate dikinase (*ppdk*), fructose-1,6-bisphosphate aldolase (*aldo*) and enolase (*eno*), which are involved in the glycolytic pathway, were obviously overexpressed. Both transaldolase (*TAL*) and transketolase (*TKL*) create a reversible link between the glycolysis and the PP pathway, which helps microbes to adapt the production of NADPH and ribose 5-phosphate in *vivo* in face of challenges (Matsushika et al., 2012). In this work, *TAL* and *TKL* were observed to have 2.26- and 2.02-fold up-regulation of expression, respectively. The TCA cycle and glycolytic pathway play important roles in furnishing intermediates and metabolic energy during microbes growth (Guan et al., 2014). As two main types of energy-transducing molecules, ATP and NADH, can generated via glycolsis and the TCA cycle (Peterson et al., 2012). As a result, it was vital to enhance energy reserves for microbes when facing the challenge of multiple stressors and genetic manipulation. The proteomics analysis showed that multiple DEPs related to central carbon metabolism were positively up-regulated to accelerate the flux through glycolysis and the TCA pathway to strengthen the accumulation of ATP. For example, in glycolysis, pyruvate phosphate dikinase and enolase, which play important roles in glycolytic flux, facilitating the conversion of phosphoenolpyruvate to pyruvate with the generation of ATP, were significantly up-regulated 3.52- and 5.09-fold, respectively (Olson et al., 2017). Additionally, the distinct up-regulation of pyruvate:ferredoxin (flavodoxin) oxidoreductase that catalyzes the formation of acetyl-CoA, the pivotal precursor of the TCA cycle, was also found.

**TABLE 3:** Classification of differential expression proteins related to central carbon metabolism.

	Proteins involved in metabolic pathways			
Protein				Mutant III/Wildtype
accession	Glycolysis	TCA cycle	PP pathway	fold change
K7RNU4	Enolase			3.52^∗^
K7RP52		2-Oxoglutarate dehydrogenase E1 component		1.70
K7RQJ5	Fructose-1,6-bisphosphate aldolase			2.24
K7RR33			Transaldolase	2.26
K7RRQ6		2-Oxoglutarate dehydrogenase E2 component		1.97
K7RTB6		Succinate dehydrogenase (ubiquinone) flavoprotein subunit		2.93
K7RTZ6		Pyruvate:ferredoxin (flavodoxin) oxidoreductase		3.32^∗^
K7RUC7	Pyruvate phosphate dikinase			5.09^∗^
K7RWT6			Transketolase	2.02
K7SJ78		Pyruvate dehydrogenase E1 component		1.71
K7SK25		Fumarate reductase flavoprotein subunit		2.94

*^∗^Fold change was significantly higher than 3.*

### Verification of Gene Expression Levels by qRT-PCRs

**FIGURE 2:**
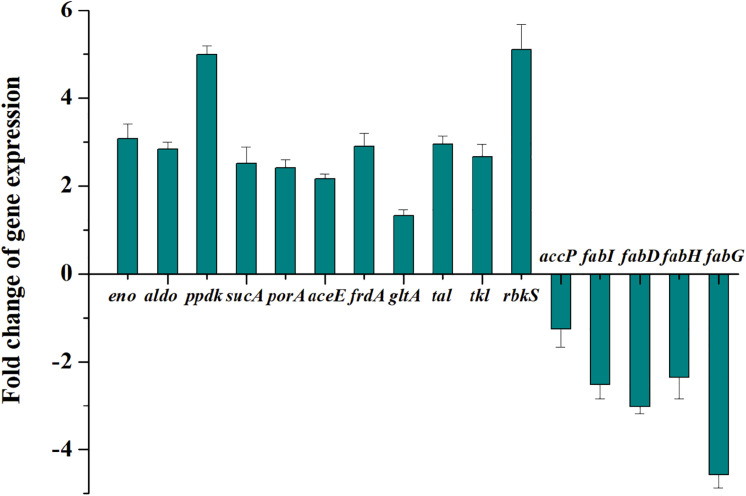
Transcription changes of 16 selected key genes related to central carbon metabolism in *P. acidipropionici* after combined metabolic and evolutionary engineering.

To validate the transcriptome and proteome data, 16 genes involved in pyruvate metabolism with relatively large differences in expression between the wild type and the mutant III were analyzed by qRT-PCR. As can be seen in Figure 2, the expression levels of the tested genes were only slightly different from the results of the omics analyses. However, the 2.52-fold up-regulation expression of *sucA* obtained from qRT-PCR was a considerably higher than that in the proteomics data, while the expression level of *porA* with 2.41-fold up-regulation through qRT-PCR analysis was lower than in the proteomics data. Moreover, the 2.51- and 3.02-fold respective down-regulations of *fabI* and *fabD*, was consistent with the expression at the transcription and protein level. Overall, the results of qRT-PCR for the 16 selected genes verified the reliability of the transcriptomic and proteomic data.

### Integrative Analyses of DEGs and DEPs Involved in Pyruvate Metabolism

**FIGURE 3:**
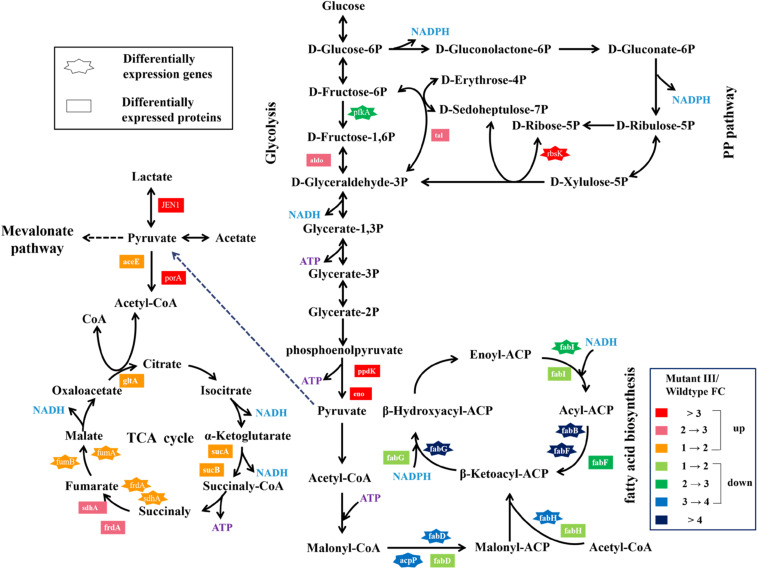
Changes of expression level of genes and proteins involved in the pyruvate metabolism and the PP pathway in *P. acidipropionici* after combined metabolic and evolutionary engineering.

Pyruvate from glucose or glycerol is a vital precursor in the metabolic pathway of propionic acid synthesis in *Propionibacterium* spp. In this study, we found that pyruvate metabolism in the mutant III was both significantly enriched from the analyses of combination of transcriptomics and proteomics compared to the wild type. A variety of metabolic pathways are involved in pyruvate metabolism, including the glycolysis pathway, fatty acid biosynthesis, TCA cycle, mevalonate pathway and so on (Fernandez et al., 2008). The differentially expressed genes and proteins related to pyruvate metabolism were both identified. As shown in Figure 3, the *pfkA* gene encoding 6-phosphofructokinase, a key gene associated with glycolysis pathway was 3.62-fold down-regulated in the mutant III relative to the wild type (Okada et al., 2015). However, at the protein level, the expression of *pfkA* gene has not been identified but the enzyme *aldo* that converts 1.6-diphosphate-D-fructose to 3-phosphate-D-glyceraldehyde increased 2.24-fold. Another two key enzymes enolase and pyruvate phosphate dikinase catalyzing the formation of phosphoenolpyruvate from glycerate-2P in the glycolysis pathway was also observed to be significantly up-regulated (Virmani et al., 2019). Generally, the absence of mRNA-protein correlation manifests that the relation between mRNA and protein is not strictly linear, but has a more inherent and complex dependence (Wang et al., 2013). The inequivalence of the identified genes in the transcriptome and proteome may be related to the dynamic regulation of cells to maintain metabolic balance. As a result, the significant up-regulation of expression of the three key genes involved in the glycolysis pathway at the protein level facilitated the considerable accumulation of ATP and pyruvate under accelerated metabolic flow from phosphoenolpyruvate, which provided abundant precursors and energy for pyruvate metabolism.

The DEGs and DEPs in the mutant III involved in TCA cycle were also investigated (Figure 3). At the transcript level, the genes *sdhA* and *frdA* were up-regulated 2.03- and 1.61-fold, compared to 2.94- and 2.93-fold up-regulation at the protein level in the mutant III. The obviously higher expression level in the proteome indicated that high protein abundance was a prerequisite for the mutant III to confront environment and DNA damage stress. Additionally, the confirmed DEGs *fumA* and *fumB*, DEPs such as *aceE*, *porA*, *gltA*, *sdhA*, *frdA* and so on all showed obvious up-regulation relative to the wild type, which implied that the TCA cycle in the mutant III was highly active. Interestingly, the quantitative superiority of DEPs compared with DEGs suggested that gene expression profiles were mainly highlighted in the form of proteome in *P. acidipropionici* after multiple stressors and genetic manipulation. The results showed a poor correlation between the quantities of these genes, which indicated a complex regulatory mechanism controlling expression both at the RNA and the protein levels.

Fatty acids are excellent sources of energy and precursors for phospholipids, sterols, sphingolipids, as secondary metabolites and signaling molecules (Janßen and Steinbüchel, 2014). In this study, we paid particular attention to the differentially expressed genes and proteins related to the most enriched fatty acid biosynthesis pathway. As can be seen in Figure 3, a total of five genes participating in fatty acid biosynthesis were down-regulated both at the mRNA and protein level to different degrees. At the RNA-Seq and proteome level, the essential gene *fabD* encoding malonyl-CoA:ACP transacylase for fatty acid biosynthesis was found to have 3.79-fold and 1.60-fold down-regulation, respectively. Malonyl-CoA:ACP transacylase can catalyze the formation of malonyl-ACP, which promotes fatty acid neogenesis and fatty acid chain elongation (Janßen and Steinbüchel, 2014). The down-regulated expression of malonyl-CoA:ACP transacylase indicated that the metabolic flux toward the synthesis of malonyl-ACP was repressed. The gene *fabH* encoding β-ketoacyl-ACP synthase III that catalyzed malonyl-ACP and acetyl-CoA units in the initial elongation step was also down-regulated 3.50- and 1.55-fold, which further restricted the metabolic flux from malonyl-ACP and led to the low accumulation of β-Ketoacyl-ACP (Handke et al., 2011). Remarkably, β-ketoacyl-ACP reductase encoded by *fabG*, which promotes the production of β-hydroxyacyl-ACP at the expense of NADPH was found to be as much as 4.17- and 1.65-fold down-regulated after the combination of metabolic and evolutionary engineering. Compared to the significant down-regulation at the RNA-Seq level (3-∼4-fold), the proteome analysis revealed lower expression profiles (1-∼2-fold), which indicated the down-regulation of the fatty acid biosynthesis process may be slowed down. Since the generation of malonyl-CoA requires ATP, the viability of microbes adopted to withstand multiple stresses and genetic manipulation is highly dependent on ATP. Based on this, the ATP in the mutant III seemed to be economically consumed for fatty acid biosynthesis to reduce the pernicious effects of environmental and DNA damage stress. The low consumption of the important precursors pyruvate and ATP, indicated that the ATP accumulation and metabolic flux to PA synthesis may be increased during fatty acid biosynthesis. Therefore, we hypothesized that the repressed metabolic flux toward fatty acid synthesis may be associated with the high yield of target product in this study.

## Conclusion

In this study, combination metabolic engineering with evolutionary engineering was first performed in *P. acidipropionici* to obtain a superior strain defined as strong robustness and high PA production capacity. The results showed that compared to the wild type, the mutant III finally obtained had a 1.5- to 3.5-fold higher growth rate, as well as a 37.1% increase of PA titer and increase of 37.8% PA productivity. Transcriptomics and proteomics data revealed that the enolase, fructose-1,6-bisphosphate aldolase and pyruvate phosphate dikinase related to the glycolysis pathway were significantly up-regulated, especially pyruvate phosphate dikinase with 5.09-fold up-regulation. In addition, seven enzymes involved in TCA cycle were observed to be obviously up-regulated, in addition to the up-regulation of transaldolase and transketolase in the PP pathway. The results indicated that the central carbon metabolism in the mutant III was up-regulated, which increased the accumulation of ATP and precursors for PA synthesis. Moreover, the down-regulations of fatty acid biosynthesis and fatty acid metabolism further implied that the consumption of energy and PA synthesis precursors was decreased. These results offer a better understanding of the mechanism of increased PA production, which lays a foundation for the construction of advanced microbial cell factories for the industrial fermentation of PA.

## Data Availability Statement

The datasets presented in this study can be found in online repositories. The names of the repository/repositories and accession number(s) can be found at: https://www.ncbi.nlm.nih.gov/, Accession No. SRR10597965 and https://www.ebi.ac.uk/pride/archive/, Accession No. PXD016616.

## Author Contributions

TL and QZ designed all the experiments. TL conducted engineered *P. acidipropionici*. YL and LZ performed physiological and fermentation profiles. HH and LJ implemented multi-omics sequencing. TL and LZ drafted this manuscript. LJ and QZ revised this manuscript. All authors read and approved the final manuscript.

## Conflict of Interest

The authors declare that the research was conducted in the absence of any commercial or financial relationships that could be construed as a potential conflict of interest.

## Footnotes

1 http://www.geneontology.org/

2 http://www.genome.jp/kegg/

## References

[B1] Aburjaile F. F. Madec M. N. Parayre S. Miyoshi A. Azevedo V. Le Loir Y. (2016). The long-term survival of *Propionibacterium freudenreichii* in a context of nutrient shortage. *J. Appl. Microbiol.* 120 432–440. 10.1111/jam.13000 26551688

[B2] Almario M. P. Reyes L. H. Kao K. C. (2013). Evolutionary engineering of *Saccharomyces cerevisiae* for enhanced tolerance to hydrolysates of lignocellulosic biomass. *Biotechnol. Bioeng.* 110 2616–2623. 10.1002/bit.24938 23613173

[B3] Asfahl K. L. Schuster M. (2018). Additive effects of quorum sensing anti-activators on *Pseudomonas aeruginosa* virulence traits and transcriptome. *Front. Microbiol.* 8:2654. 10.3389/fmicb.2017.02654 29375519 PMC5767178

[B4] Duarte J. C. Valença G. P. Moran P. J. Rodrigues J. A. R. (2015). Microbial production of propionic and succinic acid from sorbitol using *Propionibacterium acidipropionici*. *Amb. Express* 5:13. 10.1186/s13568-015-0095-6 PMC4385012 25852990

[B5] Fernandes F. A. N. Gallao M. I. Rodrigues S. (2008). Effect of osmotic dehydration and ultrasound pre-treatment on cell structure: melon dehydration. *Lwt-Food. Sci. Technol.* 1 604–610. 10.1016/j.lwt.2007.05.007

[B6] Fernandez A. Ogawa J. Penaud S. Boudebbouze S. Ehrlich D. van de Guchte M. (2008). Rerouting of pyruvate metabolism during acid adaptation in *Lactobacillus bulgaricus*. *Proteomics* 8 3154–3163. 10.1002/pmic.200700974 18615427

[B7] Guan N. Du B. Li J. Shin H. D. Chen R. R. Du G. (2018). Comparative genomics and transcriptomics analysis-guided metabolic engineering of *Propionibacterium acidipropionici* for improved propionic acid production. *Biotechnol. Bioeng.* 115 483–494. 10.1002/bit.26478 29064557

[B8] Guan N. Shin H. D. Chen R. R. Li J. Liu L. Du G. (2014). Understanding of how *Propionibacterium acidipropionici* respond to propionic acid stress at the level of proteomics. *Sci. Rep.* 4:6951. 10.1038/srep06951 PMC4223659 25377721

[B9] Guan N. Zhuge X. Li J. Shin H. D. Wu J. Shi Z. (2015). Engineering *propionibacteria* as versatile cell factories for the production of industrially important chemicals: advances, challenges, and prospects. *Appl. Microbiol. Biot.* 99 585–600. 10.1007/s00253-014-6228-z 25431012

[B10] Handke P. Lynch S. A. Gill R. T. (2011). Application and engineering of fatty acid biosynthesis in *Escherichia coli* for advanced fuels and chemicals. *Metab. Eng.* 13 28–37. 10.1016/j.ymben.2010.10.007 21056114

[B11] Huang H. Yao Q. Xia E. Gao L. (2018). Metabolomics and transcriptomics analyses reveal nitrogen influences on the accumulation of flavonoids and amino acids in young shoots of tea plant (*Camellia sinensis* L.) associated with tea flavor. *J. Agr. Food. Chem.* 66 9828–9838. 10.1021/acs.jafc.8b01995 30198713

[B12] Jantama K. Haupt M. J. Svoronos S. A. Zhang X. Moore J. C. Shanmugam K. T. (2008). Combining metabolic engineering and metabolic evolution to develop nonrecombinant strains of *Escherichia coli* C that produce succinate and malate. *Biotechnol. Bioeng.* 99 1140–1153. 10.1002/bit.21694 17972330

[B13] Janßen H. J. Steinbüchel A. (2014). Fatty acid synthesis in *Escherichia coli* and its applications towards the production of fatty acid based biofuels. *Biotechnol. Biofuels* 7:7. 10.1186/1754-6834-7-7 24405789 PMC3896788

[B14] Jiang L. Cui H. Zhu L. Hu Y. Xu X. Li S. (2015). Enhanced propionic acid production from whey lactose with immobilized *Propionibacterium acidipropionici* and the role of trehalose synthesis in acid tolerance. *Green. Chem.* 17 250–259. 10.1039/c4gc01256a

[B15] Kato Y. Ho S. H. Vavricka C. J. Chang J. S. Hasunuma T. Kondo A. (2017). Evolutionary engineering of salt-resistant *Chlamydomonas* sp. strains reveals salinity stress-activated starch-to-lipid biosynthesis switching. *Bioresour. Technol.* 245 1484–1490. 10.1016/j.biortech.2017.06.035 28624244

[B16] Lemos J. A. Nascimento M. M. Lin V. K. Abranches J. Burne R. A. (2008). Global regulation by (p) ppGpp and CodY in *Streptococcus* mutans. *J. Bacteriol.* 190 5291–5299. 10.1128/jb.00288-08 18539745 PMC2493251

[B17] Liu L. Guan N. Zhu G. Li J. Shin H. D. Du G. (2016). Pathway engineering of *Propionibacterium jensenii* for improved production of propionic acid. *Sci. Rep.* 6:19963. 10.1038/srep19963 PMC4750426 26814976

[B18] Liu L. Zhu Y. Li J. Wang M. Lee P. Du G. (2012). Microbial production of propionic acid from *propionibacteria*: current state, challenges and perspectives. *Crit. Rev. Biotechnol.* 32 374–381. 10.3109/07388551.2011.651428 22299651

[B19] Luna-Flores C. H. Stowers C. C. Cox B. M. Nielsen L. K. Marcellin E. (2018). Linking genotype and phenotype in an economically viable propionic acid biosynthesis process. *Biotechnol. Biofuels.* 11:224. 10.1186/s13068-018-1222-9 PMC6090647 30123322

[B20] Mans R. Daran J. M. G. Pronk J. T. (2018). Under pressure: evolutionary engineering of yeast strains for improved performance in fuels and chemicals production. *Curr. Opin. Biotech.* 50 47–56. 10.1016/j.copbio.2017.10.011 29156423

[B21] Matsushika A. Goshima T. Fujii T. Inoue H. Sawayama S. Yano S. (2012). Characterization of non-oxidative transaldolase and transketolase enzymes in the pentose phosphate pathway with regard to xylose utilization by recombinant *Saccharomyces cerevisiae*. *Enzyme Microbial. Tech.* 51 16–25. 10.1016/j.enzmictec.2012.03.008 22579386

[B22] Meng J. Wang B. He G. Wang Y. Tang X. Wang S. (2019). Metabolomics integrated with transcriptomics reveals redirection of the phenylpropanoids metabolic flux in Ginkgo biloba. *J. Agr. Food. Chem.* 67 3284–3291. 10.1021/acs.jafc.8b06355 30802049

[B23] Minic Z. (2015). Proteomic studies of the effects of different stress conditions on central carbon metabolism in microorganisms. *J. Proteom. Bioinform.* 8:80.

[B24] Okada K. Horii E. Nagashima Y. Mitsui M. Matsuura H. Fujiwara S. (2015). Genes for a series of proteins that are involved in glucose catabolism are up-regulated by the Hik8-cascade in *Synechocystis* sp. *PCC* 6803. *Planta* 241 1453–1462. 10.1007/s00425-015-2270-z 25732003

[B25] Olson D. G. Hörl M. Fuhrer T. Cui J. Zhou J. Maloney M. I. (2017). Glycolysis without pyruvate kinase in *Clostridium thermocellum*. *Metab. Eng.* 39 169–180. 10.1016/j.ymben.2016.11.011 27914869

[B26] Peterson C. N. Levchenko I. Rabinowitz J. D. Baker T. A. Silhavy T. J. (2012). RpoS proteolysis is controlled directly by ATP levels in *Escherichia coli*. *Genes Dev.* 26 548–553. 10.1101/gad.183517.111 22426532 PMC3315116

[B27] Purvis J. E. Yomano L. P. Ingram L. O. (2005). Enhanced trehalose production improves growth of *Escherichia coli* under osmotic stress. *Appl. Environ. Microbiol.* 71 3761–3769. 10.1128/aem.71.7.3761-3769.2005 16000787 PMC1168978

[B28] Siero J. C. Strother M. K. Faraco C. C. Hoogduin H. Hendrikse J. Donahue M. J. (2015). In vivo quantification of hyperoxic arterial blood water T-1. *Nmr. Biomed*. 28 1518–1525. 10.1002/nbm.3411 26419505 PMC4618707

[B29] Virmani R. Sajid A. Singhal A. Gaur M. Joshi J. Bothra A. (2019). The Ser/Thr protein kinase PrkC imprints phenotypic memory in *Bacillus anthracis* spores by phosphorylating the glycolytic enzyme enolase. *J. Biol. Chem.* 294 8930–8941. 10.1074/jbc.ra118.005424 30952697 PMC6552411

[B30] Wallenius J. Pahimanolis N. Zoppe J. Kilpeläinen P. Master E. Ilvesniemi H. (2015). Continuous propionic acid production with *Propionibacterium acidipropionici* immobilized in a novel xylan hydrogel matrix. *Bioresour. Technol.* 197 1–6. 10.1016/j.biortech.2015.08.037 26313629

[B31] Wang X. Salvachúa D. i Nogué V. S. Michener W. E. Bratis A. D. Dorgan J. R. (2017). Propionic acid production from corn stover hydrolysate by *Propionibacterium acidipropionici*. *Biotechnol. Biofuels* 10:200. 10.1186/s13068-017-0884-z PMC5561626 28824710

[B32] Wang Y. Y. Ren T. Cai Y. Y. He X. Y. (2013). MicroRNA let-7a inhibits the proliferation and invasion of nonsmall cell lung cancer cell line 95D by regulating K-Ras and HMGA2 gene expression. *Cancer Biother. Radio* 28 131–137. 10.1089/cbr.2012.1307 23134218

[B33] Wang Z. Jin Y. Yang S. (2015a). High cell density propionic acid fermentation with an acid tolerant strain of *Propionibacterium acidipropionici*. *Biotechnol. Bioeng.* 112 502–511. 10.1002/bit.25466 25257628

[B34] Wang Z. Lin M. Wang L. Ammar E. M. Yang S. T. (2015b). Metabolic engineering of *Propionibacterium freudenreichii* subsp. shermanii for enhanced propionic acid fermentation: effects of overexpressing three biotin-dependent carboxylases. *Process. Biochem.* 50 194–204. 10.1016/j.procbio.2014.11.012 25447642

[B35] Weimar J. D. DiRusso C. C. Delio R. Black P. N. (2002). Functional role of fatty acyl-coenzyme a synthetase in the transmembrane movement and activation of exogenous long-chain fatty acids amino acid residues within the ATP/AMP signature motif of *Escherichia coli* FadD are required for enzyme activity and fatty acid transport. *J. Biol. Chem.* 277 29369–29376. 10.1074/jbc.m107022200 12034706

[B36] Wu Q. Liu T. Zhu L. Huang H. Jiang L. (2017). Insights from the complete genome sequence of *Clostridium tyrobutyricum* provide a platform for biotechnological and industrial applications. *J. Ind. Microbiol. Biotechnol.* 44 1245–1260. 10.1007/s10295-017-1956-6 28536840

[B37] Xin Z. Liu L. Shin H. Li J. Du G. Chen J. (2014). Improved propionic acid production from glycerol with metabolically engineered *Propionibacterium jensenii* by integrating fed-batch culture with a pH-shift control strategy. *Bioresour. Technol.* 152 519–525. 10.1016/j.biortech.2013.11.063 24333145

[B38] Yang H. Wang Z. Lin M. Yang S. (2018). Propionic acid production from soy molasses by *Propionibacterium acidipropionici*: fermentation kinetics and economic analysis. *Bioresour. Technol.* 250 1–9. 10.1016/j.biortech.2017.11.016 29153644

[B39] Zhang A. Sun J. Wang Z. Yang S. T. Zhou H. (2015). Effects of carbon dioxide on cell growth and propionic acid production from glycerol and glucose by *Propionibacterium acidipropionici*. *Bioresour. Technol.* 175 374–381. 10.1016/j.biortech.2014.10.046 25459845

[B40] Zhang A. Yang S. (2009). Engineering *Propionibacterium acidipropionici* for enhanced propionic acid tolerance and fermentation. *Biotechnol. Bioeng.* 104 766–773. 19530125 10.1002/bit.22437

[B41] Zhu Z. Yang J. Yang P. Wu Z. Zhang J. Du G. (2019). Enhanced acid-stress tolerance in *Lactococcus lactis* NZ9000 by overexpression of ABC transporters. *Microb. Cell. Fact.* 18:136. 10.1186/s12934-019-1188-8 PMC6693162 31409416

