# Supplementary material for: Transcriptomics and Proteomics Analyses of the Responses of Propionibacterium acidipropionici to Metabolic and Evolutionary Manipulation
Source: Front Microbiol. 2020 Aug 13;11:1564. doi: 10.3389/fmicb.2020.01564 (PMC7438477; doi:10.3389/fmicb.2020.01564)
Supplement: Supplementary file 1 [file Table_1.doc]

**Transcriptomics and proteomics analyses of *Propionibacterium acidipropionici* with high production of propionic acid through combination metabolic with evolutionary engineering**

Tingting Liu^1,2^, Qianru Zhao^1^, Yang Li^1^, Liying Zhu^3,*^, Ling Jiang^2,*^, He Huang^4^

^1^College of Biotechnology and Pharmaceutical Engineering, Nanjing Tech University, Nanjing 210009, People’s Republic of China; ^2^College of Food Science and Light Industry, Nanjing Tech University, Nanjing 210009, People’s Republic of China; ^3^College of Chemical and Molecular Engineering, Nanjing Tech University, Nanjing 210009, People’s Republic of China; ^4^College of Pharmaceutical Science, Nanjing Tech University, Nanjing 210009, People’s Republic of China

^*^Corresponding authors.

Liying Zhu, Email: zlyhappy@njtech.edu.cn, Tel: +86-25-58139942,

College of Chemical and Molecular Engineering, Nanjing Tech University, Nanjing 210009, People’s Republic of China;

Ling Jiang, Email: jiangling@njtech.edu.cn, Tel: +86-25-58139942,

College of Food Science and Light Industry, Nanjing Tech University, Nanjing 210009, People’s Republic of China

**Table S1.** Descriptions and sources of strains, plasmids and primers.

| Strains | Descriptions/characteristics | Reference/source |
| --- | --- | --- |
| 1. *acidipropionici* | CGMCC1.2232 | CGMCC |
| E. coli DH5α | Host cells used in the cloning | Takara |
| *E. coli* S17–1 (λ pir) | Donor cells used in the conjugation | NTCC |
| Plasmids |  |  |
| pBluescriptII SK+ | f1(+) ori; Amp^R^; pUC ori | Jiang et al., 2015 |
| pJP5603 | R6K‐based suicide vector; Kan^R^ | Jiang et al., 2015 |
| pBRESP36A | shuttle vector; Ery^R^; Amp^R^; | Jiang et al., 2015 |
| pJLDH | pBluescriptII SK+ with *ldh* | This work |
| pJPOXB | pBluescriptII SK+ with *poxB* | This work |
| pBMMC | pBluescriptII SK+ with *mmc* over-expression | This work |
| Primers | Sequence(5’-3’) |  |
| *ldh*-for1 | ATATCTAGAGTCCGGATACACAGCCGTGCG  TATGAATTCGGGCTCCTCCTCGATGACGGGTCTTCTGC  ATAGAATTCTGACGTCAACAGTCGGGGGTGGAA  TATAAGCTTCGATCCGCAGACTGATCGTCTCGGAG | |
| *ldh*-rev1 |
| *ldh*-for2 |
| *ldh*-rev2 |
| *poxB*-for1 | ATATCTAGAGCTCACCGTCGGCATCGAAGGGT  TATGAATTCTGATTACCTCCGGATCCAGGTGATG  ATAGAATTCAGCCCACGGCACACCGGCCGGG  TATAAGCTTCGATGTGGTCCTCATGGCCATGGGTCAGC  ATATGATCAATGAGTCCGCGAAAGATTGGCGTTACCG  TATCCGCGGTCACGCAGGCTCGACGGTCACGTCG | |
| *poxB*-rev1 |
| *poxB*-for2 |
| *poxB*-rev2 |
| *mmc*-for1 |
| *mmc*-rev2 |

**Table S2.** Primers for genes verified through qRT-PCR.

| Genes | F-primer | R-primer |
| --- | --- | --- |
| *eno* | GAGGTTGACCTCATCACTG | CGTCCATGCCAATTTCAATTCG |
| *aldo* | TCAGCTGATCGACGCC | ATGAACACGACTCAGTTGGA |
| *ppdk* | TCACTTGTCCCTCTCGACC | ATGACTGACCAACACCGTTA |
| *sucA* | TCATCGCTGCGCGAAGG | GTCGCTGTCTGCGGGGAT |
| *porA* | ATGGAACGCACCCGGATC | TCAGGAGCGCGTCGAAGC |
| *aceE* | ATGGCCGGAGCGGAGACT | TCAGTTGGCCTCCTCGGGG |
| *frdA* | ATGTCCATTACGTCCAAA | TCACTTGTAGCTCCTCTGC |
| *gltA* | ATGGACCAGCTCGTCGATG | TCAGCCTCGCTGGGAGCGCGGG |
| *tal* | TCACTTCGCGGCGTCCAG | ATGTCCTCATCCACCGCAGC |
| *tkl* | TCACTTGGCGAGAACCTT | ATGAGAGGAACACTGACAGTG |
| *rbkS* | TCATCGGCCCTCACCCCTC | GTGAGTCAGCGCATCGTCGT |
| *acpP* | ATGTCGACGAGTGCCACC | TCAGGCCTGCTTGCGGGTGACG |
| *fabI* | ATGGGACTTCTCGACGGT | TCAGGCGCCGGTGGAATGCAG |
| *fabD* | ATGACGCAATTTGCATTTGT | TTAAAGCTCGAGCGCCGCT |
| *fabH* | ATGCCCGAGTCAACCCGC | TCAGGGGAGGACGACGACCTGG |
| *fabG* | ATGAATTTTGAAGGAAA | TCAGACCATGTACATCCCGCCG |


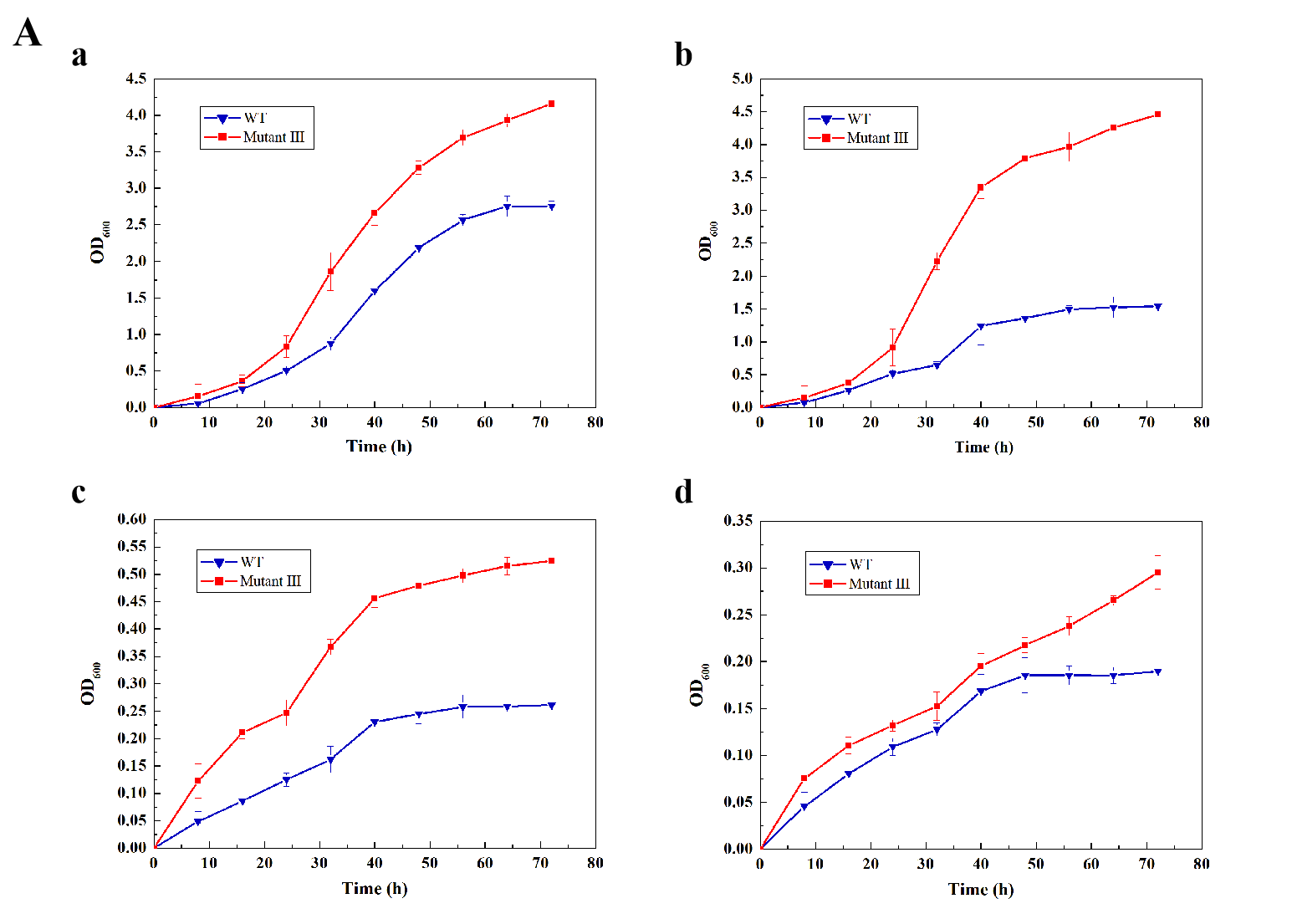

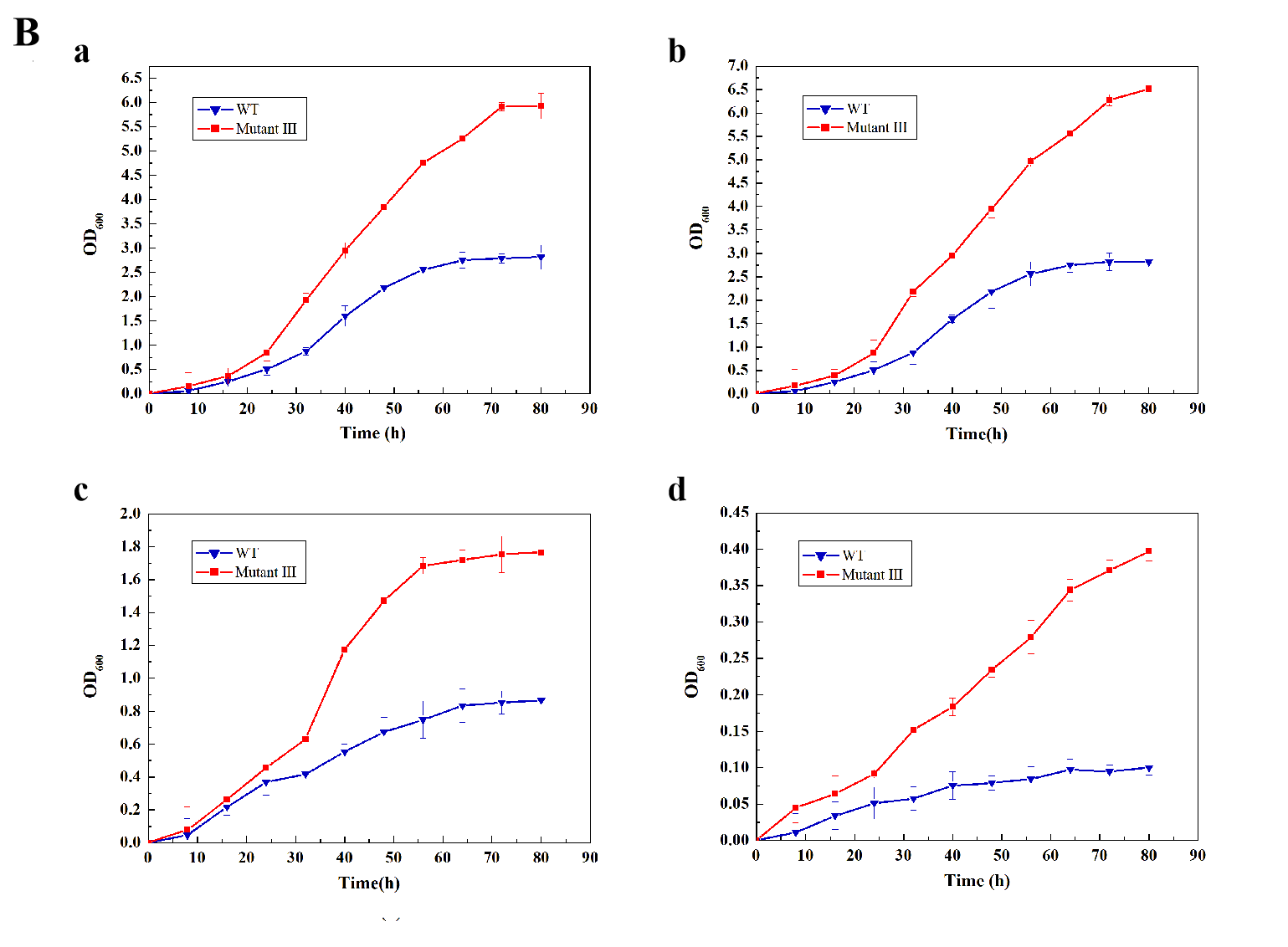


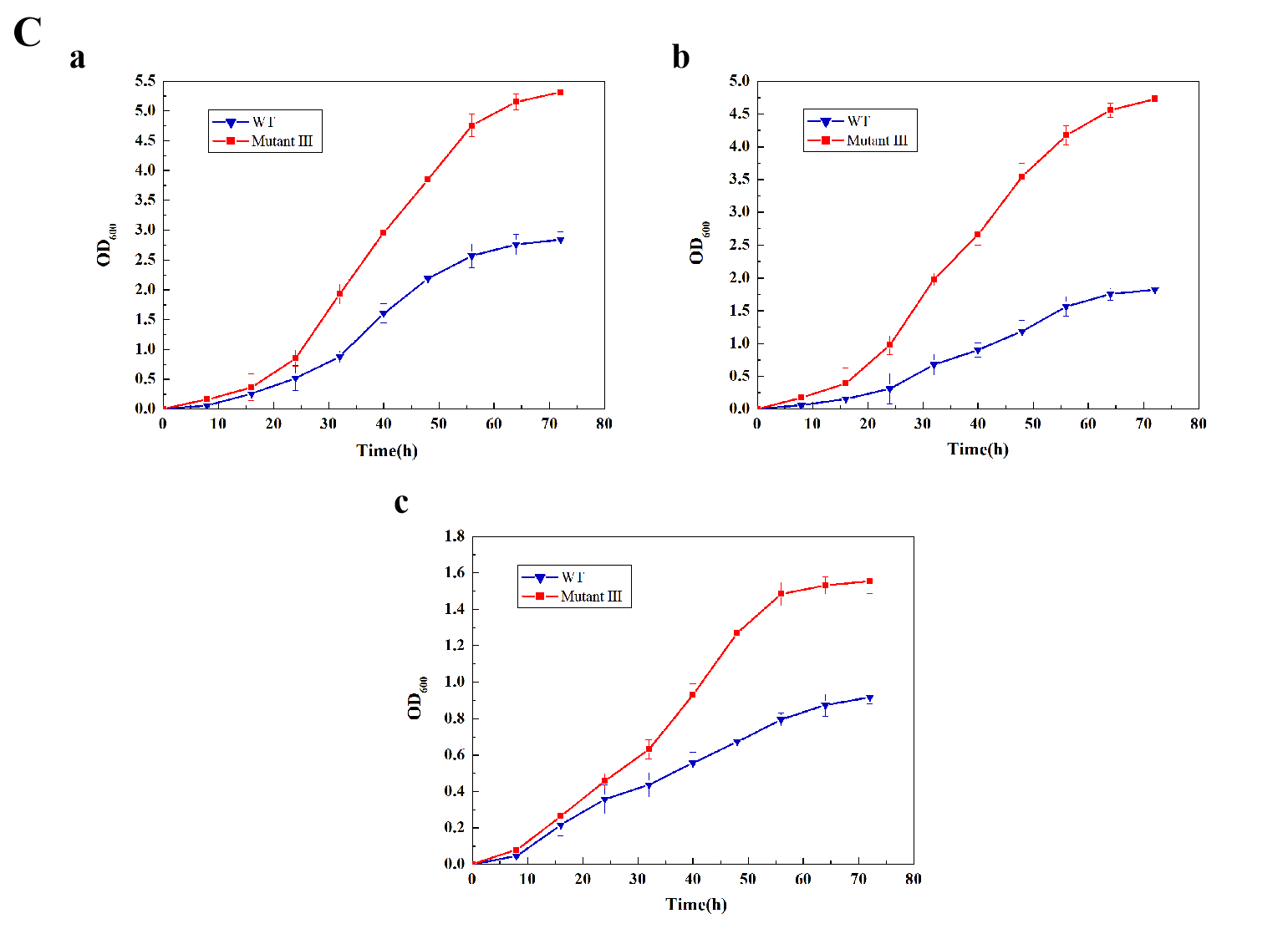


**Figure S1.** **A** Growth profiles of the wildtype and the mutant Ⅲ under different pH **a** pH 7, **b** pH 6, **c** pH 5, **d** pH 4; **B** Different glucose concentration **a** 30 g·L^-1^, **b** 60 g·L^-1^, **c** 90 g·L^-1^, **d** 120 g·L^-1^; **C** Different oxygen content **a** 0%, **b** 10%, **c** 21%.


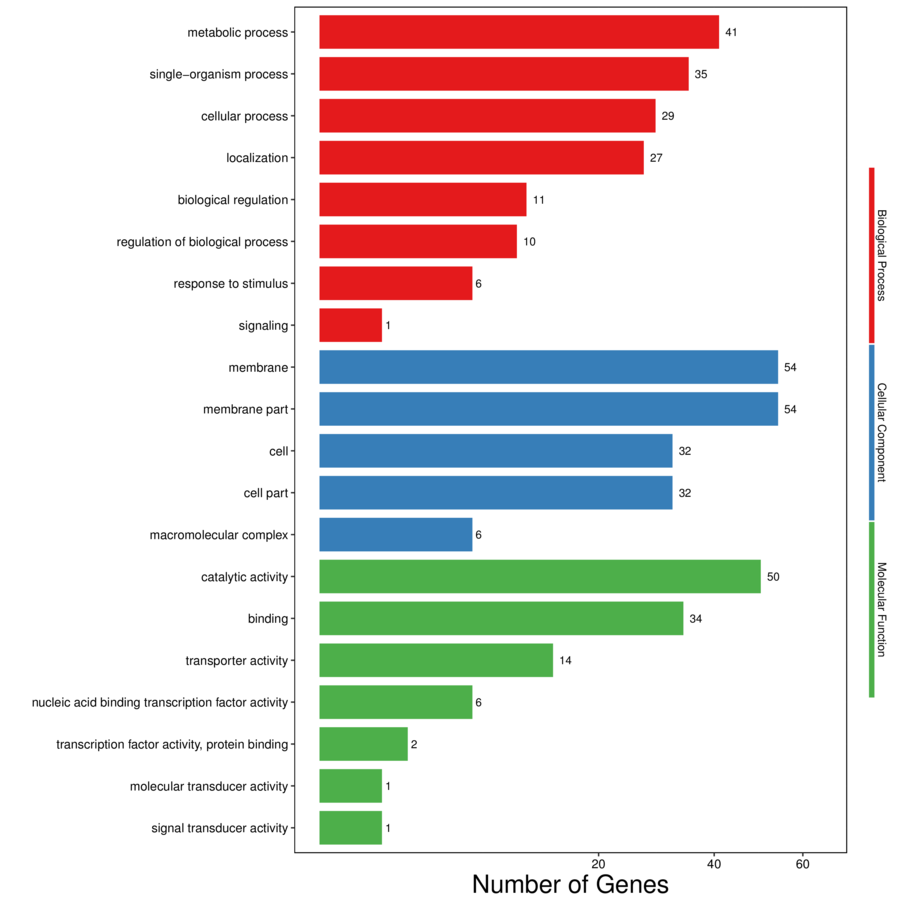


**Figure S2.** The GO functional analysis of the DEGs in the mutant Ⅲ compared to the wild type.


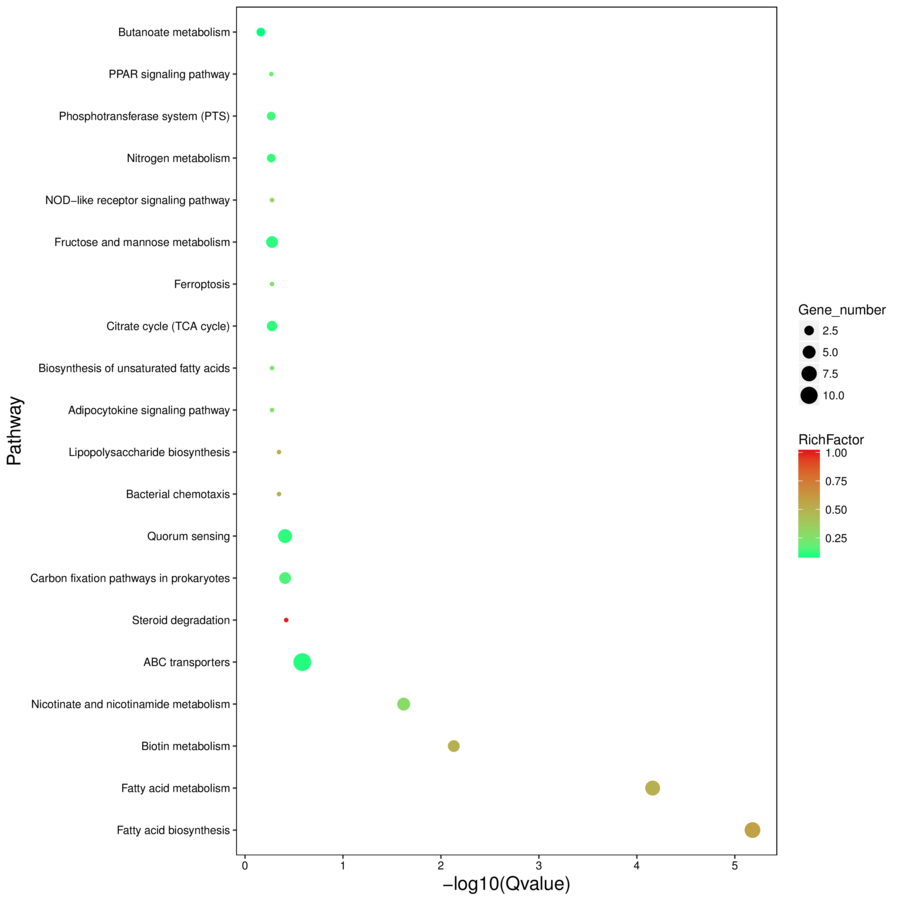


**Figure S3.** The KEGG pathway enrichment analysis of the DEGs in the mutant Ⅲ compared to the wild type.


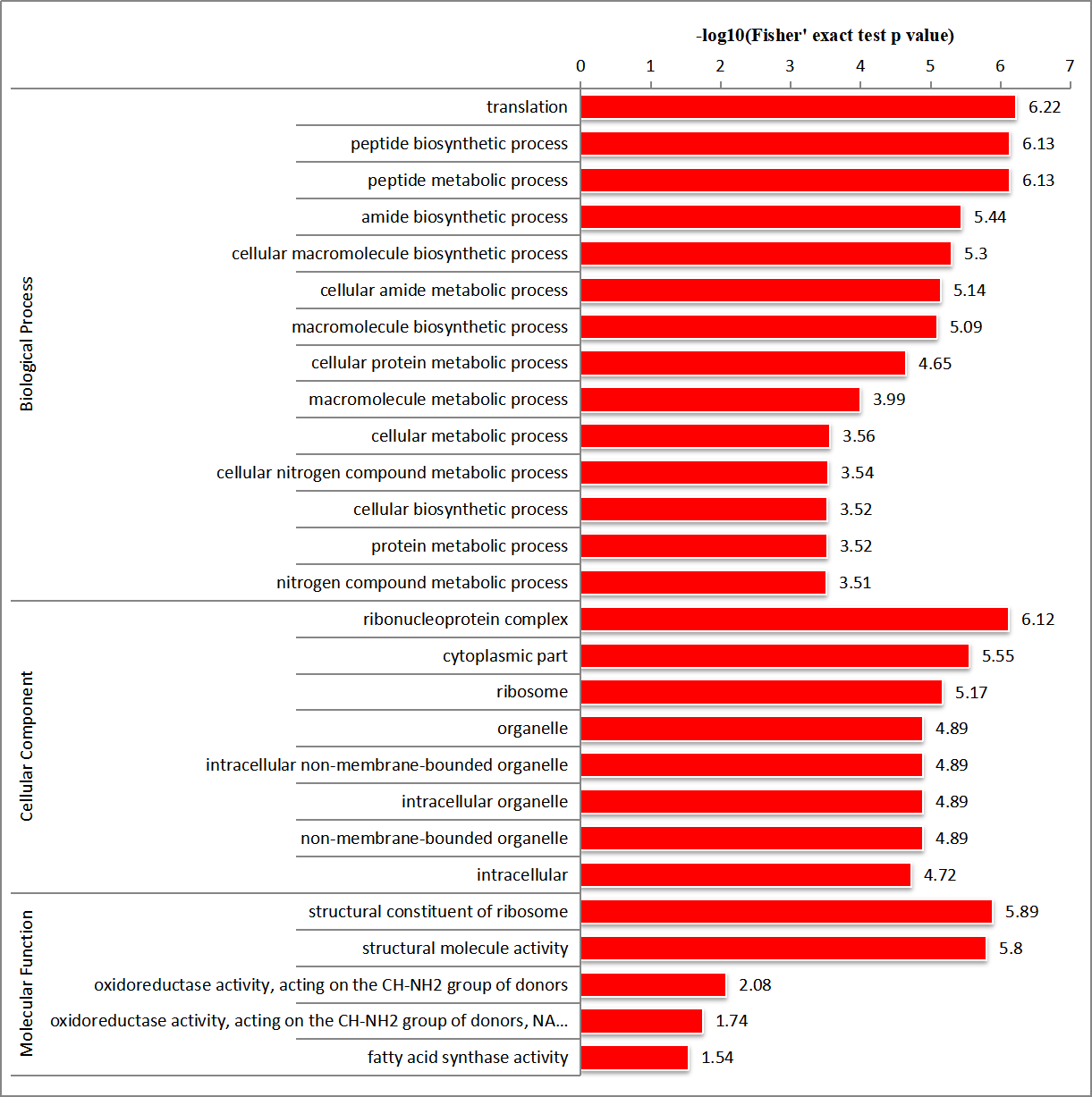


**Figure S4.** The GO functional analysis of the DEPs from the mutant Ⅲ compared to the wild type.


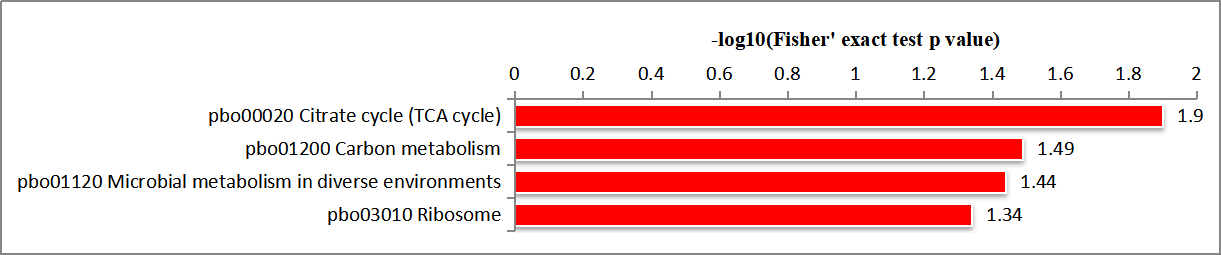


**Figure S5.** The KEGG pathway enrichment analysis of the DEPs from the mutant Ⅲ compared to the wild type.
